# A Hybrid CNN–MLLM Architecture for Image-Based Nutrition Estimation and Advisory Insulin Decision Support in Type 1 Diabetes

**DOI:** 10.3390/nu18132205

**Published:** 2026-07-07

**Authors:** Jean Chrinot Velombe, Sema Bayraktar, Adnan Kavak, Muhammad Jamil, Alpaslan Burak İnner, Gautam Srivastava, Hossein Fotouhi

**Affiliations:** 1 Department of Computer Engineering, Kocaeli University, 41001 İzmit, Türkiye; 235112002@kocaeli.edu.tr (J.C.V.); jamil138.amin@gmail.com (M.J.); binner@kocaeli.edu.tr (A.B.İ.); 2 Wireless Information and Intelligent Systems (WINS) Research Center, Kocaeli University, 41001 Kocaeli, Türkiye; bayraktar.sema41@gmail.com; 3 Department of Computer Science and Engineering, Sogang University, Seoul 04107, Republic of Korea; 4 Research Center for Interneural Computing, China Medical University, Taichung 40402, Taiwan; 5 Centre for Research Impact & Outcome, Chitkara University Institute of Engineering and Technology, Chitkara University, Rajpura 140401, Punjab, India; 6 Department of Computer Science and Computer Engineering, Mälardalen University (MDU), 72123 Västerås, Sweden

**Keywords:** Type 1 diabetes, food image recognition, nutrition estimation, insulin bolus decision support, mobile health

## Abstract

Background/Objectives: Accurate estimation of meal composition from food images can support safer and more reliable insulin bolus decision-making for individuals with Type 1 diabetes. Existing food recognition and nutrition estimation systems are often designed for general dietary logging and do not directly integrate food analysis with personalized insulin therapy parameters. Methods: This study presents an image-based nutrition estimation and insulin decision-support module developed within the AI-assisted Diabetes Care (AIDCARE) platform. The proposed system uses a convolutional neural network (CNN) to classify food items from a single meal image, and retrieves reference nutritional values from a food composition database. A separate multimodal large language model (MLLM)-based estimation component is then used to estimate portion size, allowing carbohydrate and nutrient values to be scaled according to the observed serving. Results: A curated food image dataset containing 40 food categories was used to evaluate three CNN architectures: ResNet50, Inception V3, and EfficientNet-B0. EfficientNet-B0 achieved the best classification performance, with 94.91% validation accuracy, 95.55% precision, 94.87% recall, and 94.90% F1-score. The portion-estimation component achieved an MAE of 12.27 g and an RMSE of 15.11 g. The estimated carbohydrate value is combined with user-specific clinical parameters, including the insulin-to-carbohydrate ratio and insulin sensitivity factor, to generate advisory bolus guidance. To support safety, the system requires user confirmation or correction of the recognized food category and estimated portion before insulin guidance is displayed. Conclusions: The proposed system is intended for advisory decision support only and is not designed to replace clinical judgment or autonomous insulin delivery systems.

## 1. Introduction

People with Type 1 diabetes must monitor daily food intake and estimate appropriate meal-related insulin doses to maintain stable blood glucose levels [1,2]. In real-world settings, manual approaches such as visual portion estimation or food weighing are commonly used. However, these methods are time-consuming and error-prone because they rely on subjective judgment. Such errors may reduce the accuracy of insulin dosing and make blood glucose control more difficult [3,4,5].

In recent years, advances in artificial intelligence (AI), deep learning (DL), and computer vision (CV) have provided practical approaches for reducing human error in dietary assessment. These methods can support food recognition, nutrient estimation, and semi-automated dietary tracking from food images [6,7]. Some studies have investigated 3D reconstruction approaches for estimating food volume and nutrient content from food images [8]. However, reconstruction-based methods often require specialized hardware, depth information, reference objects, or multiple images captured from different angles. These requirements limit their practical use in everyday mobile health settings, especially for individuals who need fast, simple meal logging with a standard smartphone camera.

### 1.1. Research Gap and Motivation

Several AI-powered applications have been developed for calorie counting and nutrition logging. However, most existing systems focus on food recognition or nutrient estimation as standalone tasks and do not directly connect image-based food analysis with personalized insulin decision support for individuals with Type 1 diabetes. Furthermore, many existing approaches require specialized hardware, depth sensors, multiple images, or complex user interaction, limiting their practical adoption in routine diabetes self-management. Consequently, there remains a need for a clinically supervised and mobile-friendly framework capable of estimating food category, portion size, nutrient content, and advisory insulin guidance from a single meal image while maintaining appropriate safety constraints.

### 1.2. Research Objective

The objective of this study is to develop and evaluate an AI-assisted nutrition estimation and advisory insulin decision-support framework within the AIDCARE platform. The proposed framework combines food recognition, portion estimation, nutrient analysis, and patient-specific insulin guidance while preserving clinician oversight through a human-in-the-loop safety mechanism. The system is intended to support both individuals with Type 1 diabetes and healthcare professionals by providing rapid and explainable nutritional assessment from a single meal image.

In this study, we propose an image-based nutrition estimation and insulin decision-support module within the AIDCARE platform. The system allows users to upload a single meal image, predicts the food category, estimates the portion size, retrieves reference nutritional values, and computes advisory bolus guidance using user-specific clinical parameters. The system incorporates a human-in-the-loop mechanism that allows users and healthcare professionals to review, confirm, or correct food and portion estimates before insulin guidance is displayed.

This design preserves the system’s advisory role, supports safer decision-making in diabetes self-management, and enables clinician oversight via the AIDCARE healthcare platform.

(1) $$J\left(\theta ,\phi \right)=\frac{1}{N}\sum_{i=1}^{N}\left[L_{\mathrm{cls}}\left(y_{i},f_{\theta }\left(I_{i}\right)\right)+\lambda L_{\mathrm{wt}}\left(w_{i},h_{\phi }\left(I_{i}\right)\right)\right]$$

Equation (1) provides a conceptual formulation of the coupled image-based nutrition estimation task. For each meal image $I_{i}$, the system aims to identify the food category $y_{i}$ and estimate the corresponding portion weight $w_{i}$. The function $f_{\theta }\left(\cdot \right)$ denotes the CNN-based food classification model with parameters θ, whereas $h_{\phi }\left(\cdot \right)$ denotes the portion-estimation component with parameters ϕ. The classification loss $L_{\mathrm{cls}}$ measures food-category prediction error, while the weight-estimation loss $L_{\mathrm{wt}}$ measures portion-estimation error. The coefficient λ controls the relative contribution of portion-estimation error in the overall task formulation. In the deployed AIDCARE implementation, food recognition and portion estimation are implemented as a hybrid two-stage pipeline rather than a single jointly trained network.

To implement this research, we selected the most suitable classifiers, including ResNet50, EfficientNet-B0, and Inception V3, and trained them using images captured by actual users. After identifying the food category, the system retrieves carbohydrate content and other nutritional information and scales them according to the estimated portion size. The resulting values contribute to bolus calculations that incorporate the individual’s Insulin-to-Carbohydrate Ratio (ICR) and Insulin Sensitivity Factor (ISF).

The system is intended to assist both patients and clinicians in meal-related insulin decision-making. Users must always verify and either accept or adjust the identified food category and nutrient estimates before any insulin advice is provided. This human-in-the-loop feature maintains user control over critical decisions. It should be noted that Equation (1) expresses the conceptual joint objective of the proposed methodology. In the deployed AIDCARE implementation, however, food recognition and portion estimation are not realized as a single jointly trained network. Instead, the practical system follows a hybrid two-stage pipeline in which a CNN-based classifier predicts the food category and a separate multimodal model estimates portion size.

### 1.3. Scientific Contributions and Novelty

The scientific contributions of this work are summarized as follows:

- A hybrid CNN–MLLM framework that combines food classification and image-based portion estimation from a single meal photograph.
- An automated nutrient estimation pipeline that retrieves reference nutritional values and scales them according to the estimated serving size.
- An advisory insulin decision-support module that incorporates patient-specific clinical parameters, including the insulin-to-carbohydrate ratio and insulin sensitivity factor.
- Integration of the complete workflow into the AIDCARE healthcare ecosystem, enabling clinician supervision through a dedicated web-based management platform.
- A human-in-the-loop safety mechanism that requires user verification before insulin recommendations are displayed.

The proposed framework differs from existing CNN-based food recognition systems, which primarily focus on food identification, and from multimodal nutrition-estimation approaches that typically provide dietary analysis without direct integration into diabetes management workflows. In contrast, the proposed system links food recognition, portion estimation, nutrient analysis, clinician-configurable treatment parameters, and advisory insulin support within a unified healthcare platform. Unlike fully automated insulin recommendation approaches, the framework maintains clinical oversight through mandatory user verification and clinician supervision, thereby improving safety, transparency, and practical applicability in real-world diabetes care. Furthermore, although the present study focuses on Turkish food categories, the underlying architecture is not cuisine-specific. The food classification component can be retrained using local food datasets, while the nutrient estimation and insulin advisory modules remain applicable across different healthcare environments. This design supports future adaptation to other countries, dietary cultures, and regulatory settings.

### 1.4. Paper Organization

The remainder of this paper is organized as follows. Section 2 reviews existing studies related to food recognition, nutrition estimation, and AI-assisted diabetes support systems. Section 3 presents the architecture of the AIDCARE platform and its major components. Section 4 describes the proposed methodology, including dataset preparation, food classification, portion estimation, nutrient scaling, and insulin advisory calculations. Section 5 reports the experimental results and discusses their implications, limitations, and clinical relevance. Finally, Section 6 concludes the paper and outlines future research directions.

## 2. Related Work

One of the most critical aspects of Type 1 diabetes self-management is the ability to accurately estimate a carbohydrate meal due to the direct relationship between the amount of carbohydrate consumed and the amount of insulin required [9,10]. Many patients use either manual (counting) or visual estimation of meal portions in practice. While they are common approaches, they are also subjective and subject to significant estimation errors, especially for mixed dishes, irregular portion sizes and meals outside the home. This can result in underestimation or overestimation of insulin doses, leading to suboptimal glucose control. In response to this drawback, researchers have turned to automatic dietary analysis methods based on computer vision and deep learning [11,12] to overcome it.

Early research focused mainly on food image classification, which involved assigning meal images into one or more food categories. Convolutional neural networks (CNNs) have significantly improved the performance of food recognition, and many architectures, such as ResNet, Inception, and EfficientNet, are widely used for food recognition applications in dietary assessment, as in [13,14]. These studies showed that CNN-based models could learn robust visual representations in realistic scenarios with cluttered backgrounds, visually similar food categories and different lighting. Food classification, however, is not sufficient to provide information about the quantity of food consumed or the nutrient content of the food for clinical nutritional support.

Another big research thrust is food portion estimation and quantitative nutrient assessment. Various studies have explored the use of geometric modeling, fiducial markers, depth sensors, multi-view reconstruction, and reference objects to estimate food volume and quantity from images [15,16,17]. While these techniques can enhance quantitative estimation accuracy, several require controlled acquisition conditions, specialized hardware or multiple images taken from varying perspectives. This restricts their use in real-world health care settings, where users generally take only one photo with the smartphone camera.

This has made lightweight single-image methods with sufficiently accurate portion estimation without requiring extra sensors very interesting.

Recently, multimodal large language models (MLLMs) have been investigated in food-related reasoning scenarios, such as ingredient detection, nutritional description, and approximate estimation of food quantities from images [18]. Additionally, related work indicates that egocentric instruction tuning extends the capabilities of MLLMs in visual understanding by aligning the interpretation of orientation with the user’s view, which is applicable to image-based tasks with consistent spatial interpretation [19]. The ability of MLLMs to better understand meal context and produce human-readable nutritional estimates is more flexible than that of purely CNN-based pipelines. However, the current MLLM-based nutrition systems are mostly used for estimating calories, logging diets, or explaining meals. The assimilation of their integration into clinically relevant insulin decision-making workflows is still limited.

In parallel with developments in computer vision, many nutrition applications for mobile and web platforms have been developed to aid in dietary monitoring, calorie counting, meal logging, and the provision of personalized nutrition guidance [20,21,22]. These systems are useful in enhancing adherence, improving nutrition awareness, and making self-tracking easier. Most, however, focus on overall health, weight loss, or general nutrition monitoring instead of on the needs of people with T1D.

Most current systems stop at the stage of food recognition or nutrient estimation and do not incorporate patient-specific insulin therapy parameters (e.g., insulin-to-carbohydrate ratio (ICR) and insulin sensitivity factor (ISF). This means they provide nutrition information, but not directly into the decision-making process of meal bolus. This is representative of an important difference between general nutrition assessment applications and diabetes decision-support systems.

Table 1 shows a summary of representative works on food recognition, nutrient estimation, and diabetes support systems in the image-based domain. This comparison highlights their datasets, methods, goals, achieved performance, and key limitations.

The comparison in Table 1 indicates that previous studies have made important contributions to food recognition, carbohydrate estimation, and diabetes self-management support. However, most approaches address these components independently. Existing food recognition systems often lack quantitative nutrient estimation, while nutrition estimation solutions typically do not incorporate patient-specific insulin therapy parameters. Similarly, diabetes-support applications frequently rely on manual meal entry and fail to leverage recent advances in computer vision and multimodal artificial intelligence.

The proposed AIDCARE framework addresses this gap by integrating CNN-based food classification, single-image portion estimation using a multimodal model, database-driven nutrient scaling, patient-specific insulin advisory calculations, clinician-configurable treatment parameters, and a human-in-the-loop verification mechanism within a unified healthcare platform. Unlike approaches that require multiple images, depth sensing, or 3D reconstruction, the proposed system is designed to operate from a single meal photograph captured using a standard smartphone camera. Furthermore, unlike conventional calorie-counting applications, the framework extends beyond nutrition logging to support advisory insulin decision-making under clinician supervision. This combination of image-based nutritional analysis and personalized diabetes decision support distinguishes the proposed approach from existing systems and motivates the research presented in this paper.

## 3. System Overview and Hybrid AI Architecture

**Figure 1:**
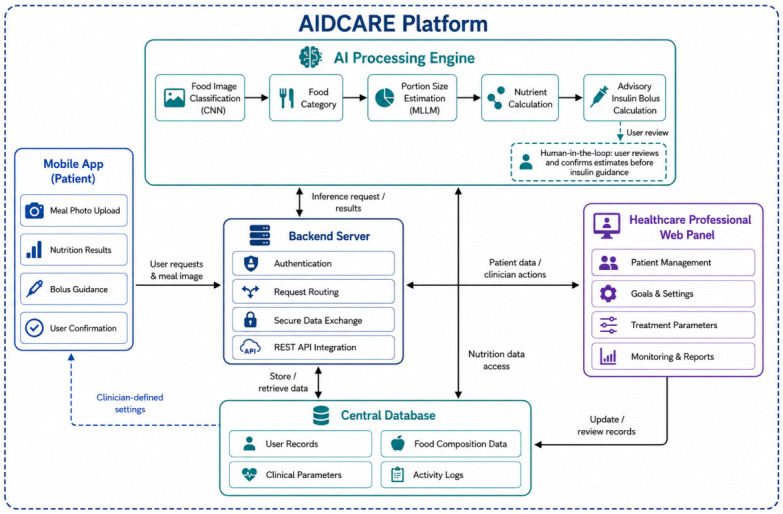
Overall architecture of the proposed system within the AIDCARE platform.

We developed the decision-support system for image-based nutrition analysis as a central module of the AIDCARE platform; this broader digital health tool is designed to assist individuals in managing diabetes in their everyday lives. Within this methodology, our research emphasizes assessing dietary intake and providing guidance on insulin boluses. The system integrates food recognition from images with user-tailored nutritional information and relevant clinical parameters. Figure 1 illustrates the full architecture of this hybrid AI method along with its integration into the overall AIDCARE ecosystem.

### 3.1. Platform-Level System Architecture

The proposed system has four major modules, including a mobile app, a web panel for healthcare professionals, a backend server and an AI processing engine. All these modules are connected using REST APIs and exchange data with a central database for persistent storage and access. The mobile app is developed for patients with Type 1 diabetes and serves as the point of interaction for end users. The user can upload a single food image, which is processed by the AI engine for classification and nutrient estimation. Depending on the input image, the application shows estimated nutrient breakdowns. The results are shared with the patient, and once the user approves, they can request a bolus recommendation. The backend server serves as the primary module connecting the other modules. It is responsible for user authentication, secure data exchange between modules, request routing, and sending storage requests to the central database. The AI engine is responsible for core computational tasks such as food image classification by category, portion size estimation, and calculation of essential carbohydrate values. The results are used for bolus calculations. Mathematically:

(2) $$\hat{\pi }\left(I\right)=\mathrm{softmax}\;\left(f_{\theta }\left(I\right)\right)$$

here *I* is the input image, $f_{\theta }\left(\cdot \right)$ represents the CNN class prediction function with parameters θ, and $\hat{\pi }\left(I\right)$ is the predicted probability distribution among all food categories. Here *K* denotes the number of food categories in the dataset, in our case, K=40).

(3) $$\hat{y}=\arg\max_{k}{\hat{\pi }}_{k}\left(I\right)$$

here $\hat{y}$ is the predicted category with the highest probability among all possible classes.

(4) $$\hat{w}=h_{\phi }\left(I\right)$$

where $h_{\phi }\left(\cdot \right)$ denotes the portion-size estimation model and $\hat{w}$ is the raw estimated food weight in grams derived from the input image.

The component-based architecture makes it simple to connect with other parts of the AIDCARE solution. The AIDCARE platform offers additional features, including body measurements, exercise planning, diabetes-related education, a gamification module and HbA1c risk prediction.

### 3.2. Diet Management Module and User Interaction Workflow

The diet management module is at the heart of our contribution and part of the AIDCARE solution. It allows patients with Type 1 diabetes to document meals and receive personalized guidance on insulin boluses based on estimated carbohydrate intake. After the image is uploaded from a smartphone camera, the AI engine first applies a CNN model (EfficientNet) to determine the food category. That classification then pulls corresponding reference nutritional data from an established food composition database.

(5) $$y^{*}=C_{y}\left(\hat{y}\right),\;w^{*}=C_{w}\left(\hat{w}\right)$$

where $C_{y}\left(\cdot \right)$ and $C_{w}\left(\cdot \right)$ denote the mandatory user confirmation operators for food category and portion size, respectively. During review, the user may either accept the predicted values or revise them, resulting in the final approved outputs $y^{*}$ and $w^{*}$.

Next, a multimodal analysis component processes the same image to predict portion size. Carbohydrate and other nutrient values are computed by adjusting the database entries according to this estimate.

The total carbohydrate estimate $\hat{C}$ is computed by scaling database carbohydrate values using the confirmed portion size as defined in Equation (8).

Finally these figures combine with the user’s personal parameters notably the Insulin-to-Carbohydrate Ratio (ICR) and Insulin Sensitivity Factor (ISF) to produce bolus dosage suggestions. These remain strictly advisory to assist decision-making.

(6) $$u_{\mathrm{food}}=\frac{\hat{C}}{\mathrm{ICR}}$$

$u_{\mathrm{food}}$ is the insulin amount required to cover meal carbohydrates, $\hat{C}$ is the estimated carbohydrate intake, and ICR is the insulin-to-carbohydrate ratio defined by clinicians.

**Figure 2:**
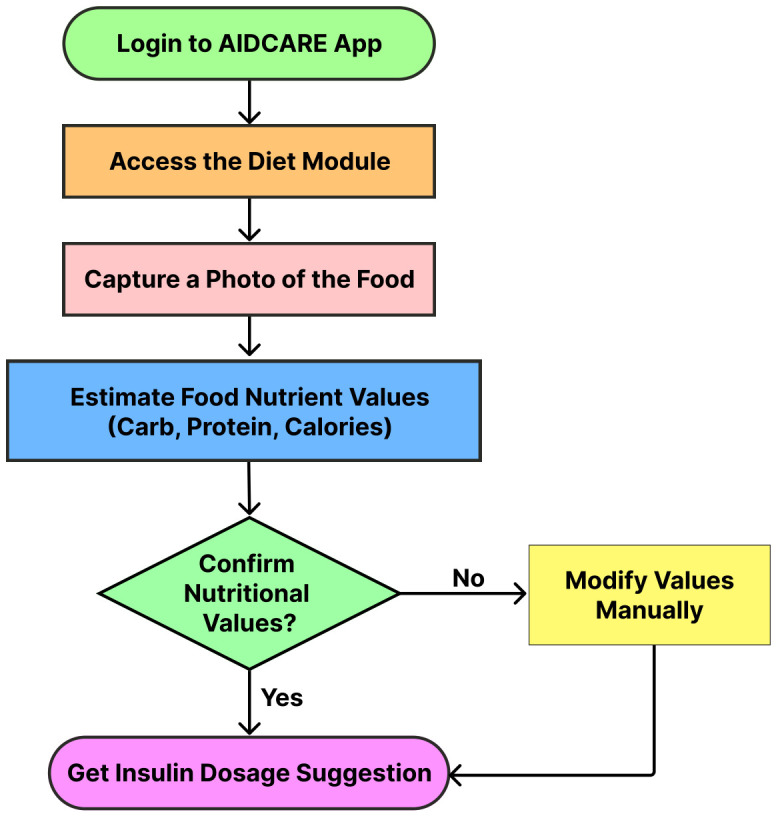
Workflow of the diet management module for image-based nutrition analysis and insulin bolus decision support.

For safety, we built in a mandatory validation step: before any bolus advice appears, users must review and either confirm or revise the detected food category and nutrient calculations. This human-in-the-loop design keeps users accountable for the most important details and reinforces the system’s role as supportive rather than autonomous. The full sequence of interactions in the diet management module is shown in Figure 2.

### 3.3. Healthcare Professional Web Panel

In the AIDCARE platform, a dedicated web-based panel was developed for healthcare professionals, including dietitians and clinicians, to support patient management and personalized diabetes care. Through this interface, healthcare professionals can manage patient records, configure treatment parameters, define blood glucose targets, monitor nutritional goals, and track patient progress over time. The web panel also provides access to appointments, messaging, AI-assisted suggestions, reports, and online consultation features.

Personalized treatment settings, including blood glucose targets, meal-specific nutrition goals, insulin-to-carbohydrate ratios (ICR), and other clinician-defined parameters, can be configured through the platform and synchronized with the patient mobile application. This enables treatment plans to be updated in real time according to individual patient requirements, activity levels, and observed glycemic trends.

**Figure 3:**
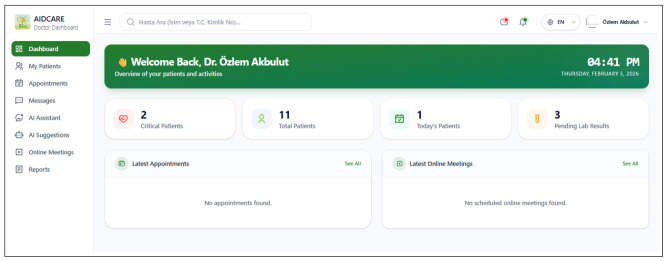
Overview of the AIDCARE clinician dashboard, showing sidebar navigation, patient statistics, and activity summaries.

The clinician dashboard provides a consolidated overview of patient statistics, alerts, recent activities, and platform usage, enabling healthcare professionals to monitor and manage multiple patients in a single interface efficiently. Figure 3 presents an overview of the AIDCARE clinician dashboard.

The clinician dashboard is integrated with the nutrition management workflow and enables healthcare professionals to review patient information, configure treatment parameters, and supervise the use of AI-generated nutritional recommendations. This integration supports clinician oversight and facilitates individualized diabetes management within the broader AIDCARE ecosystem.

### 3.4. Security, Privacy, and Regulatory Considerations

The proposed AIDCARE framework was designed with security and privacy considerations appropriate for healthcare environments. Food images, nutritional records, and patient-specific treatment parameters are transmitted through authenticated channels and stored within controlled healthcare infrastructure. User access is restricted through role-based authorization mechanisms that distinguish between patients, nutrition professionals, and clinicians. Furthermore, the platform follows a human-in-the-loop design in which all insulin recommendations remain advisory and subject to user verification before any action is taken. To protect patient privacy, only the information required for nutritional assessment and decision support is processed by the system. The framework is designed to support compliance with healthcare data protection principles, including data minimization, access control, auditability, and secure data management practices. Since the current system functions as a clinical decision-support tool rather than an autonomous treatment system, final treatment decisions remain under the control of patients and clinicians.

### 3.5. Deployment Considerations and Computational Complexity

The proposed framework was designed for practical deployment within the AIDCARE healthcare platform. During inference, the computational workload is dominated by the CNN-based food classifier and the multimodal portion estimation module. Among the evaluated architectures, EfficientNet-B0 was selected because it achieved the highest classification performance while maintaining lower computational complexity than larger CNN models. The overall inference workflow consists of four sequential stages: food classification, portion estimation, nutrient scaling, and insulin advisory calculation. The nutrient scaling and insulin advisory components require only lightweight arithmetic operations and contribute negligible computational overhead compared with image processing stages. Consequently, the deployment cost is primarily determined by image analysis. From a practical perspective, the system is intended for real-time clinical use where users upload a single meal image through a mobile device. The architecture therefore avoids computationally expensive requirements such as 3D reconstruction, depth sensing, multiple image acquisition, or specialized hardware. This design reduces deployment complexity and supports scalability within routine healthcare environments.

## 4. Materials and Methods

### 4.1. Dataset and Pre-Processing

We assembled a custom dataset of high-resolution food images to train and evaluate the food classification models in a Turkish food context. The dataset sources included publicly available recipe websites and real-world images captured by users, enabling it to represent dishes commonly consumed in everyday Turkish diets.

The collected images represent substantial real-world variability in food appearance and acquisition conditions. The dataset includes photographs captured under different indoor and outdoor lighting conditions, varying camera viewing angles, different plate and bowl types, diverse food presentation styles, background clutter, and variations in serving arrangements. These variations were intentionally retained during dataset curation to improve model robustness and better reflect practical smartphone-based meal logging scenarios encountered by individuals with Type 1 diabetes. The image collection process combined publicly available Turkish food recipe resources with real-world user-contributed meal photographs. This approach allowed the dataset to capture both professionally presented food images and naturally occurring meal photographs acquired under everyday conditions.

After careful curation and annotation, the final dataset contained 22,070 food images across 40 distinct Turkish food categories representing typical daily intake patterns. Although the number of images varied across categories, the complete curated dataset was retained for model development and evaluation to preserve the diversity of real-world food appearances. To mitigate class imbalance, stratified sampling was used during dataset splitting, and data augmentation was applied only to the training subset. The dataset was randomly partitioned into training (80%) and validation (20%) subsets while preserving the class distribution across all food categories.

**Figure 4:**
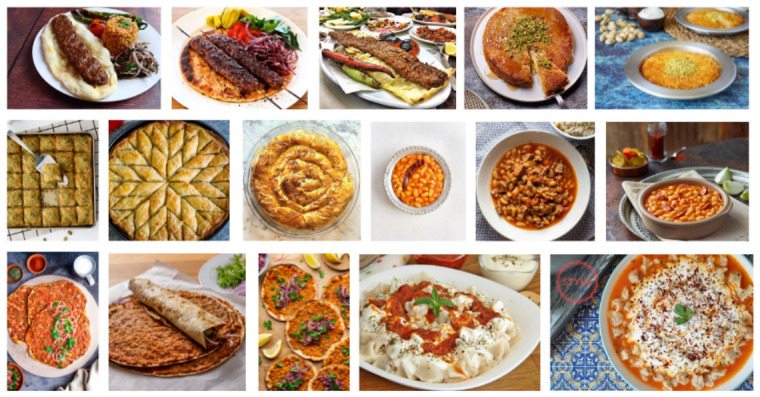
Selected examples from the food image dataset.

A single hold-out validation strategy was adopted in this study, and k-fold cross-validation was not performed. The validation subset remained completely unseen during training and was used exclusively for model evaluation. To avoid experimental data leakage, the dataset was partitioned before model training, and augmentation was applied only to the training images after the split. Therefore, no augmented version of a validation image was included in the training data. This separation ensured that the reported performance metrics were computed on unseen images and reflected model generalization rather than memorization. A subset of dataset samples is visualized in Figure 4.

To improve model generalization and robustness, we applied data augmentation techniques using the torchvision.transforms library in Python (Version 3.12.3). These techniques included random horizontal and vertical flips, random rotations within ±20°, color jittering for brightness, contrast, and saturation, and random resizing and cropping to an input resolution of 224×224 pixels.

All images in the dataset were normalized using ImageNet [27] statistics, with mean values of 0.485, 0.456, and 0.406 and standard deviation values of 0.229, 0.224, and 0.225, respectively. This normalization step was applied to make the input images compatible with the pre-trained CNN architectures used in this study, namely EfficientNet-B0, ResNet50, and Inception V3. Matching the expected input distribution enables more effective transfer learning and allows models to leverage learned visual features while reducing distribution shift.

### 4.2. Food Image Classification Models

To determine the most suitable model for food category recognition in our Turkish-context dataset, we evaluated three well-established convolutional neural network (CNN) architectures: ResNet50 [28], EfficientNet-B0 [29], and Inception V3 [30]. These were chosen because they have consistently demonstrated strong performance on large-scale image classification benchmarks and have already been successfully used in various food image analysis tasks [31]. We applied transfer learning starting from weights pre-trained on ImageNet1K. During fine-tuning, the convolutional feature extraction layers remained frozen to preserve the general visual representations learned from ImageNet. Only the final fully connected classification layer was replaced and trained from scratch to fit our 40-class food recognition problem.

(7) $$L_{\mathrm{cls}}=-\sum_{k=1}^{K}y_{k}\log{\hat{\pi }}_{k}$$

$L_{\mathrm{cls}}$ denotes the cross-entropy classification loss, *K* is the number of food classes, $y_{k}$ is the ground-truth label, and ${\hat{\pi }}_{k}$ is the predicted probability for class *k*. All models used cross-entropy loss and were optimized with the Adam algorithm. We trained consistently with a starting learning rate of 0.001, a batch size of 32, and 10 epochs. To prevent overfitting and improve convergence, a ReduceLROnPlateau scheduler dynamically reduced the learning rate whenever validation loss stopped improving.

#### 4.2.1. ResNet50

**Figure 5:**
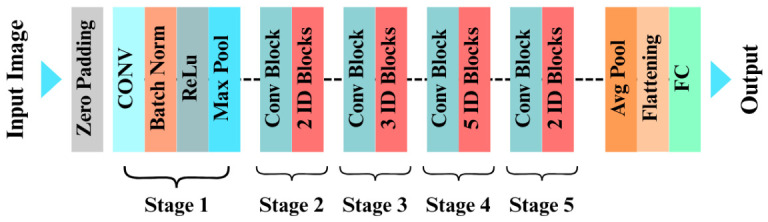
Architecture of the ResNet50 model.

ResNet50 [28] is a 50-layer deep convolutional network that relies on residual (shortcut) connections to address the challenges of training very deep architectures. The complete architecture is shown in Figure 5.

#### 4.2.2. EfficientNet-B0

**Figure 6:**
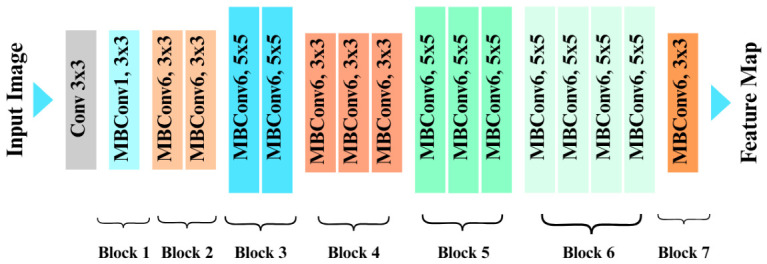
Architecture of the EfficientNet-B0 model.

EfficientNet-B0 [29] employs compound scaling to balance depth, width, and resolution, resulting in strong performance with low computational cost. The overall architecture of this model is shown in Figure 6.

#### 4.2.3. Inception V3

**Figure 7:**
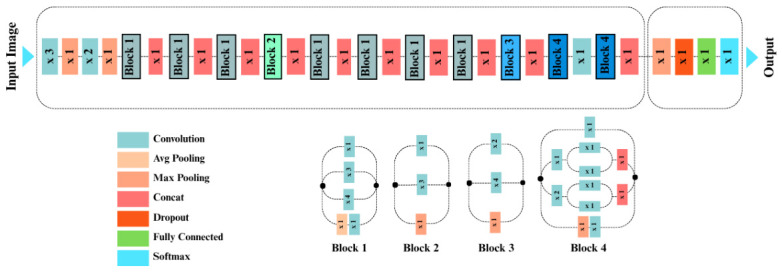
Architecture of the Inception V3 model.

Inception V3 [30] utilizes parallel convolutional branches to capture multi-scale spatial features efficiently. Figure 7 shows the architecture of Inception V3 model.

### 4.3. Hybrid AI-Based Portion and Nutrient Estimation

**Figure 8:**
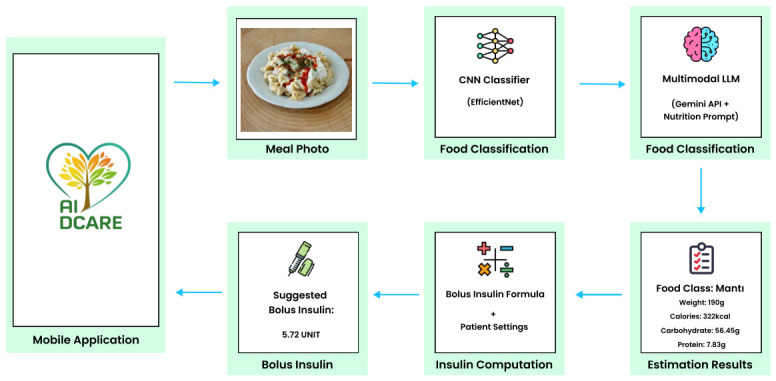
Pipeline of the hybrid nutrient estimation and insulin dosage guidance system.

Our hybrid approach combines CNN-driven food classification with a multimodal AI component to derive both portion size and nutritional estimates directly from a single food photograph, as illustrated in Figure 8.

Once the food category is identified, the portion size is estimated and used to scale reference nutritional values obtained from the food database.

(8) $$\hat{C}=\frac{w^{*}}{100}\;r_{\mathrm{carb}}\left(y^{*}\right)$$

(9) $$\Delta C_{b}=\frac{r_{\mathrm{carb}}\left(y^{*}\right)}{100}\;\Delta w,\;|\Delta C_{b}|\;\leq \frac{r_{\mathrm{carb}}\left(y^{*}\right)}{100}\;\left|\Delta w\right|$$

To make the effect of portion-estimation uncertainty explicit, we additionally consider the first-order propagation of weight error into carbohydrate estimation. The above relation shows that the carbohydrate error grows linearly with the food-specific carbohydrate density and the portion-estimation error. $\hat{C}$ is the estimated total carbohydrate amount in grams, $w^{*}$ is the confirmed portion weight, and $r_{\mathrm{carb}}\left(y^{*}\right)$ is the carbohydrate value per 100 g for the confirmed food category. Table 2 shows the example carbohydrate calculations using standard portion sizes for selected food categories.

### 4.4. Insulin Bolus Decision Support Calculations

Meal-related insulin is computed from the estimated carbohydrate intake as follows:

(10) $$u_{\mathrm{food}}=\frac{\hat{C}}{\mathrm{ICR}}$$

(11) $$\Delta u_{\mathrm{food}}=\frac{\Delta C_{b}}{ICR}=\frac{r_{\mathrm{carb}}\left(y^{*}\right)}{100\;ICR}\;\Delta w\;$$

(12) $$|\Delta u_{\mathrm{food}}|\;\leq \frac{r_{\mathrm{carb}}\left(y^{*}\right)}{100\;ICR}\;\left|\Delta w\right|$$

Combining Equation (8) with the meal bolus rule yields a direct sensitivity relation between portion-estimation error and meal-related insulin uncertainty. This formulation makes the safety interpretation of portion-estimation accuracy more transparent. Here $u_{\mathrm{food}}$ is the insulin dose for meal carbohydrates, $\hat{C}$ is total carbohydrates in grams, and ICR is the insulin-to-carbohydrate ratio. Correction insulin is calculated to adjust for deviations from target glucose:

(13) $$u_{\mathrm{corr}}=\max\;\left(0,\frac{\mathrm{CBG}-\mathrm{TBG}}{\mathrm{ISF}}\right)$$

where CBG is the current blood glucose, TBG is the target blood glucose, and ISF is the insulin sensitivity factor. The max(0,·) term prevents negative correction insulin values when CBG<TBG.

(14) $$W\left(CBG\right)=\left\{\begin{array}{cc} 1, & CBG<TBG\\ 0, & CBG\geq TBG \end{array}\right.$$

Since Equation (13) suppresses negative correction insulin, it is useful to define an explicit low-glucose warning indicator. This preserves the advisory character of the system while making the safety logic mathematically explicit. The total advisory insulin bolus is then:

(15) $$u_{\mathrm{total}}=u_{\mathrm{food}}+u_{\mathrm{corr}}$$

(16) $$u_{\mathrm{safe}}=\max\;\left(0,\min\{u_{\max},\;u_{\mathrm{food}}+u_{\mathrm{corr}}\}\right)$$

To reflect clinician-defined treatment limits more explicitly, the advisory bolus can also be written in constrained form, where $u_{\max}$ denotes the maximum recommended bolus configured in the clinical settings panel.

$u_{\mathrm{total}}$ represents the total insulin bolus suggested by the system before user confirmation.

### 4.5. Evaluation Protocol

Food classification performance was evaluated using accuracy, precision, recall, and macro-averaged F1-score. The balanced dataset was split into training and validation sets using stratified sampling with an 80%/20% ratio to preserve class distribution. For the multi-class classification setting, precision, recall, and F1-score were computed using a one-vs-rest strategy and then macro-averaged across all food categories. Portion-weight estimation performance was evaluated using mean absolute error (MAE) and root mean squared error (RMSE). All experiments were conducted in a GPU-accelerated environment using the same training configuration across the evaluated CNN architectures to ensure a consistent comparison.

## 5. Results and Discussion

The experimental evaluation was designed to assess the proposed framework from multiple complementary perspectives. First, food classification performance was evaluated using standard classification metrics to determine the effectiveness of the CNN models in recognizing food categories. Second, portion estimation accuracy was analyzed to assess the reliability of the multimodal component responsible for serving-size estimation. Third, nutrient estimation and insulin advisory calculations were examined to evaluate the practical applicability of the framework within the AIDCARE workflow. Together, these analyses provide a comprehensive assessment of the individual components and the overall effectiveness of the proposed image-based nutrition estimation and insulin decision-support framework.

### 5.1. Food Classification Performance

For the food classification task, we evaluated three convolutional neural network (CNN) architectures: ResNet50 [28], EfficientNet-B0 [29], and Inception V3 [30]. Exact category recognition is an essential step towards reliable carbohydrate estimation. We fine-tuned all the models using transfer learning. To quantitatively evaluate classification performance, we used standard metrics; for the multi-class setting (K=40), precision, recall, and F1-score are computed in a one-vs-rest manner and reported as macro-averages across classes.

(17) $$\mathrm{Accuracy}=\frac{TP+TN}{TP+TN+FP+FN}$$

TP, TN, FP, and FN denote true positives, true negatives, false positives, and false negatives, respectively, and Accuracy measures the overall proportion of samples correctly classified.

(18) $$\mathrm{Precision}=\frac{TP}{TP+FP}$$

Precision indicates how many of the predicted food categories are correct.

(19) $$\mathrm{Recall}=\frac{TP}{TP+FN}$$

Recall measures how many true food categories the model successfully identifies.

(20) $$\mathrm{F1-Score}=2\cdot \frac{\mathrm{Precision}\cdot \mathrm{Recall}}{\mathrm{Precision}+\mathrm{Recall}}$$

F1-score is the harmonic mean of precision and recall, and is a balanced performance measure. The best overall performance was achieved by EfficientNet-B0, with a validation accuracy of 94.91%. It is competitive with others because of the joint optimization of the depth, width and resolution of the compound, which enables highly efficient detection of discriminative features even with a relatively small number of parameters. The model was quite reliable for many categories such as *Lahmacun* that are clearly visible. Compared with the following, Inception V3 was the next-closest with 91.67% validation accuracy. The Inception modules, in their multi-scale approach, were found to be effective at extracting detailed textures and the spatial context of the image. It performed well on the small number of food items with distinctive appearances, such as *Adana kebabı*, and poorly on items with similar appearances, such as *Mantı*. ResNet50 achieved 87.96% validation accuracy and was only slightly lower in recognizing visually homogeneous items like *Tarhana soup*, while showing stable performance. The comparison of Accuracy, precision, recall and F1-score between the two is shown in Table 3. The alternatives were always outperformed by efficientNet-B0, which was the backbone of choice for downstream nutrient estimation and bolus support.

**Figure 9:**
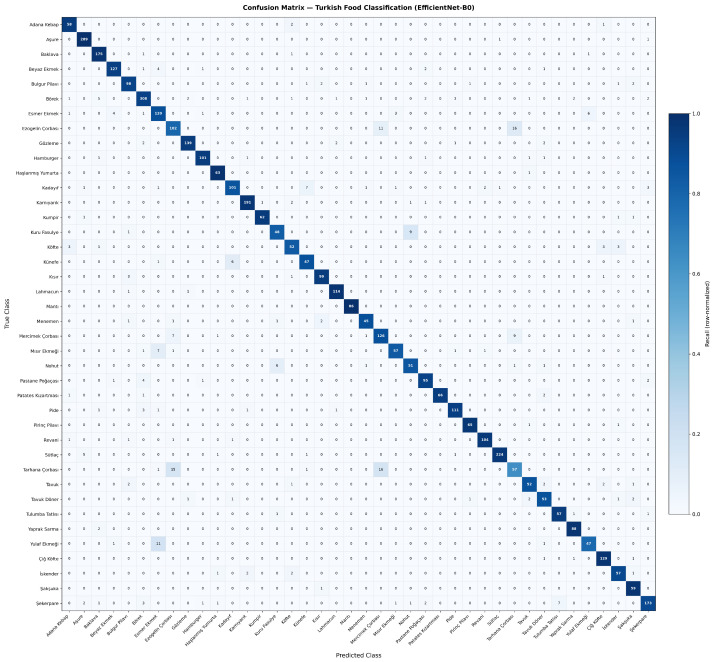
Confusion matrix of the EfficientNet-B0 classifier on the validation set.

The confusion matrix in Figure 9 provides a detailed view of class-level classification performance. Most food categories were recognized with high accuracy, indicating strong discriminative capability of the EfficientNet-B0 architecture. Misclassifications primarily occurred among visually similar dishes that share common ingredients, textures, or presentation styles. The confusion matrix confirms the robustness of the proposed classifier and supports the aggregate performance metrics reported in Table 3.

### 5.2. Gemini-Based Food Weight Estimation

We evaluated the Gemini multimodal large language model (MLLM) for food portion weight estimation using mean absolute error (MAE) and root mean square error (RMSE), defined as:

(21) $$\mathrm{MAE}=\frac{1}{N}\sum_{i=1}^{N}\left|w_{i}-{\hat{w}}_{i}\right|$$

$w_{i}$ is the true food weight, ${\hat{w}}_{i}$ is the estimated weight, and *N* is the number of test samples.

(22) $$\mathrm{RMSE}=\sqrt{\frac{1}{N}\sum_{i=1}^{N}{\left(w_{i}-{\hat{w}}_{i}\right)}^{2}}$$

**Figure 10:**
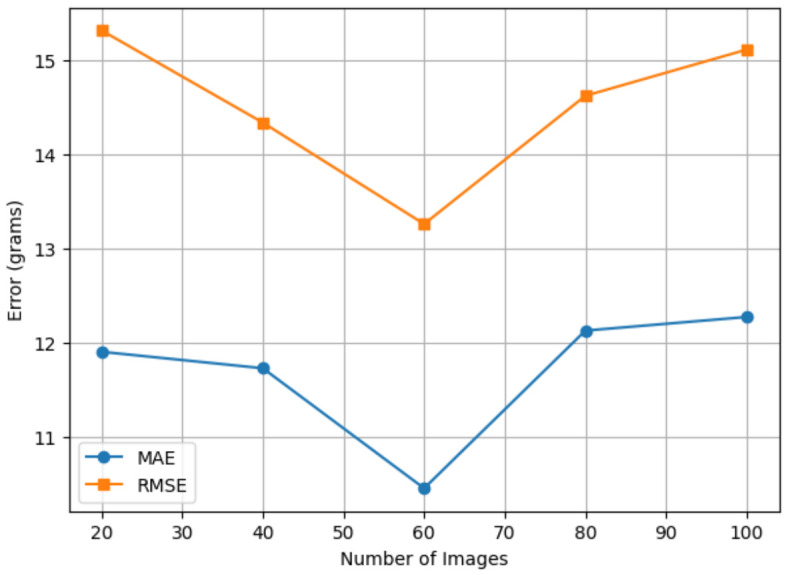
Weight estimation performance versus dataset size.

**Figure 11:**
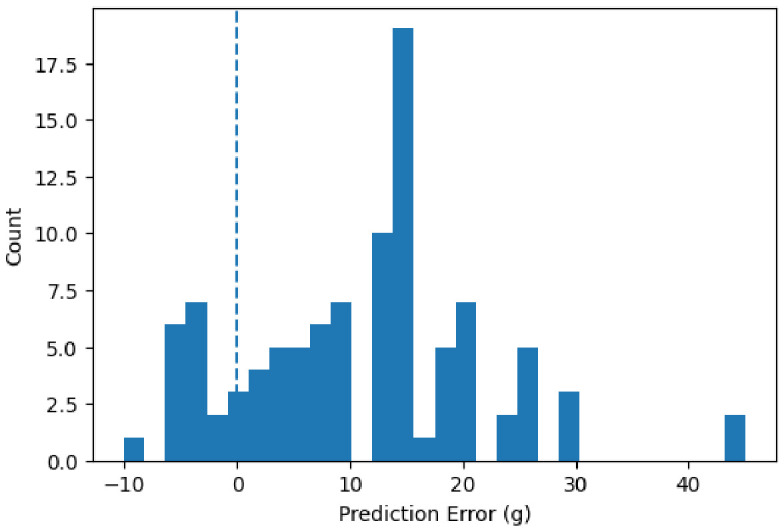
Weight estimation error distribution.

RMSE penalizes larger estimation errors more strongly by squaring the difference between true and estimated weights. Across the test set, the model achieved an MAE of 12.27 g and an RMSE of 15.11 g, indicating that most estimates were within a practically acceptable range for real-world smartphone images. Figure 10 shows that estimation accuracy remains stable across varying dataset sizes, while Figure 11 illustrates a symmetric error distribution centered near zero. These results highlight the inherent uncertainty of single-image portion estimation and justify the system’s mandatory user confirmation step.

### 5.3. System Deployment and End-to-End Evaluation on the AIDCARE Platform

The complete AI-assisted pipeline was integrated into the AIDCARE platform and evaluated under realistic usage contexts. From an end-to-end perspective, the final insulin recommendation can be explained as a function of estimated carbohydrates and clinical parameters:

(23) $$u_{\mathrm{total}}=\frac{\hat{C}}{\mathrm{ICR}}+\max\;\left(0,\frac{\mathrm{CBG}-\mathrm{TBG}}{\mathrm{ISF}}\right)$$

$\hat{C}$ is the estimated carbohydrate amount, ICR is the insulin-to-carbohydrate ratio, CBG is current blood glucose, TBG is target blood glucose, and ISF is the insulin sensitivity factor.

**Figure 12:**
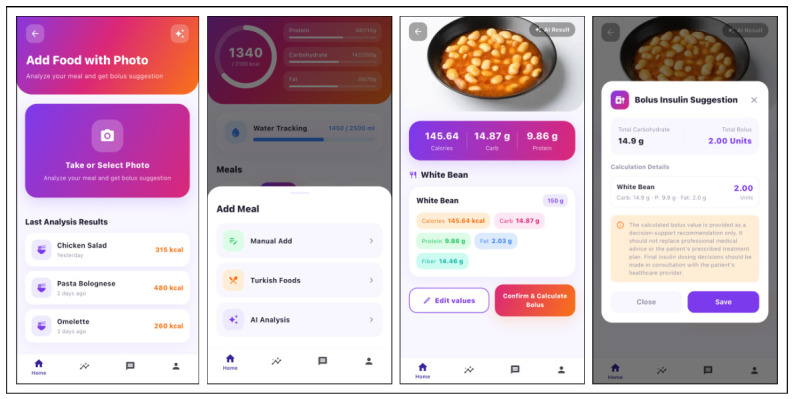
Mobile application nutrition module workflow showing food photo upload, AI-based food recognition, nutrient estimation, and advisory insulin bolus decision-support results.

Figure 12 demonstrates the deployed mobile workflow for food image upload, nutrient estimation, and advisory insulin decision support. In the illustrated example, a meal of *White Bean* was correctly classified; its portion was estimated at 150 g, resulting in 14.87 g of carbohydrates and a suggested bolus of 2.00 units. The mandatory user confirmation step ensures that all insulin guidance remains advisory and under the control of both users and clinicians.

### 5.4. Discussion

The hybrid AI-based decision support system integrates food category recognition, nutrient estimation, and AI-assisted insulin dosing recommendations to enable personalized blood sugar management for people with Type 1 diabetes. The experimental results show that deep learning models are effective at distinguishing meal categories from a single food image; this can serve as a practical basis for automated carbohydrate estimation and insulin decision support. EfficientNet-B0 achieved the highest overall accuracy of 94.91%, an F1-score of 94.90%, a recall of 94.87%, and a validation precision of 95.55%. EfficientNet-B0 showed significant improvements in predicting while achieving lower computational complexity as compared to ResNet50 and Inception V3. After food recognition, the amount of food was estimated using a multimodal large language model (Gemini Image API), enabling the system to obtain individual nutrient estimates from a single picture of a meal.

The framework will help people reduce the burden of manual carbohydrate counting and make more consistent insulin dosing decisions around meals from a clinical perspective. The system allows patients to get quick nutrient estimates from a picture without granting the system complete autonomy, with clinicians still able to provide advice. The architecture used in the present study is not cuisine-specific, although the categories used were based on Turkish foods. The food classification module can be fine-tuned using datasets from other regional cuisines, and the nutrient estimation and insulin advisory can be applied in various healthcare settings. Thus, the proposed framework could be applied to other countries and health care systems by incorporating local food data, nutrition databases, and local regulations.

One of the most important factors is the impact of errors in carbohydrate estimation on insulin dose recommendations in any image-based diabetes support system. The error in weight estimation was approximately 1–5 g of carbohydrate variation for a 15 g error in the dataset as a whole, depending on the food composition. If the portion estimation error for foods is about 15 g per 100 g of food, then the deviation of carbohydrate content is about 3 g per 100 g of food. If the insulin-to-carbohydrateinsulin-to-carbohydrate ratio (ICR) is 1:10 g/U, this mistake would be equivalent to adjusting insulin by about 0.3 units. This size is usually within the clinically accepted ranges for bolus dosing adjustments in a person’s diabetes control.

The AIDCARE platform also displays the food recognition and estimation results for the user’s review and confirmation or correction before suggesting an insulin dose, further enhancing safety. This human-in-the-loop approach is consistent with best practices in AI clinical decision support, and mitigates the risk of occasional prediction errors.

The following mathematical modelling interpretation is intended as a first-order advisory model for the present bolus formulation. It does not specifically consider insulin-on-board, physical activity, delayed effect of fat/protein, continuous glucose monitoring trends, stress, illness and other dynamic physiological factors that could affect the glucose effect following a meal. Thus, these proposed equations are meant to explain, not to serve as a complete treatment model.

Several limitations should be acknowledged. Although the dataset contains variability in lighting conditions, viewing angles, and plating styles, highly heterogeneous mixed-meal scenarios remain underrepresented. Furthermore, the current evaluation focused on technical and system-level validation rather than prospective patient outcomes. The AIDCARE platform has already been deployed at Kocaeli Research Hospital, where nutrition professionals currently use the clinical web panel. Following completion of planned patient trials, future work will evaluate glycemic outcomes, including HbA1c, time in range, postprandial glucose excursions, hypoglycemia frequency, adherence, and user acceptance. Future versions will also incorporate additional physiologically relevant insulin-dosing factors, including insulin-on-board, physical activity, continuous glucose monitoring trends, delayed fat/protein effects, stress, and illness.

## 6. Conclusions

This study presented an applied AI-based decision-support system for image-based nutrition estimation and advisory guidance for insulin bolus dosing in Type 1 diabetes self-management. Our proposed hybrid system combined CNN-based food recognition with vision-based volume estimation. The system provides reliable carbohydrate calculations based on an image captured with a mobile phone. It does not depend on resource-intensive 3D reconstruction or on multiple images. Such features make the system ideal for smart healthcare mobile applications, especially in diverse nutritional environments such as Turkish cuisine. We tested multiple DL models, and among them, EfficientNet-B0 achieved the best results with 94.91% validation accuracy, 95.55% precision, 94.87% recall, and 94.90% F1-score. After recognizing food categories, portion sizes were estimated using MLLM (Gemini Image API). This method produced sufficiently precise carbohydrate estimates, with an MAE of around 10–15 g. We sent all the information to the nutritionist dashboard to support a human-in-the-loop mechanism, keeping the bolus guidance purely advisory and ensuring compliance with clinical standards for decision support, rather than relying on fully automated processes.

Future research will focus on prospective clinical validation of the AIDCARE framework through patient trials conducted at Kocaeli Research Hospital to assess outcomes including HbA1c, time in range, postprandial glucose excursions, treatment adherence, and user acceptance. We also plan to expand the dataset to include more diverse food categories and more complex mixed-meal scenarios to improve the model’s generalization further. In addition, future versions of the system will incorporate physiologically relevant factors such as insulin-on-board, physical activity, continuous glucose monitoring trends, stress, illness, and delayed fat/protein effects to provide more personalized insulin advisory support. Finally, the framework will be adapted and evaluated using region-specific food datasets and nutritional databases to support deployment across different cuisines and healthcare environments.

## Figures and Tables

**Table 1:** Comparison of representative recent image-based food analysis and diabetes-support studies.

Study	Dataset/Setting	Method	Main Objective	Performance	Main Limitation
Alfonsi et al. [23]	Youth with Type 1 diabetes	Image-recognition mobile application	Carbohydrate counting support	High usability and clinical acceptance	Limited integration with clinician-supervised insulin decision support
Chotwanvirat et al. [24]	75,232 Thai food images	Deep learning food detection and weight estimation	Automatic carbohydrate estimation	Top-1 accuracy 80.9%	Cuisine-specific; no personalized insulin support
Qi et al. [18]	Multimodal nutrition datasets	MLLM-based nutrition reasoning	Ingredient understanding and nutrition estimation	Improved contextual food interpretation	Focused on nutrition analysis rather than diabetes decision support
Nogay et al. [25]	Turkish cuisine food dataset	CNN-based food classification and portion prediction	Food recognition and portion estimation	80% classification accuracy	No nutrient scaling or insulin advisory module
AlBabtain et al. [26]	Adults with Type 1 diabetes	Mobile carbohydrate counting and bolus calculator	Glycemic management support	Improved time-in-range by 5.03%	Requires manual meal entry; no image-based food analysis
**This Study**	Turkish food image dataset	Hybrid CNN–MLLM framework within AIDCARE	Food recognition, portion estimation, nutrient scaling, and advisory insulin support	94.91% classification accuracy; 12.27 g portion MAE	Prospective clinical validation remains future work

**Table 2:** Example carbohydrate calculations using standard portion sizes for selected food categories.

Food Category	Portion Size (g)	Carb/100 g (g)	Total Carb/Portion (g)
Mantı	190	29.71	56.45
Lahmacun	150	21.51	32.27
White beans	70	29.42	20.59
Yaprak sarma	130	17.81	23.16
Mercimek çorbası	300	8.28	24.85
Tarhana soup	250	4.99	12.47
Baklava	160	49.36	78.97
Künefe	120	45.75	54.90
Adana kebabı	151	1.06	1.60
Börek	200	35.83	71.67

**Table 3:** Food classification performance of different CNN models.

Model	Accuracy ↑	Precision ↑	Recall ↑	F1-Score ↑
ResNet50	87.96%	85.48%	84.20%	84.27%
Inception V3	91.67%	91.92%	91.62%	91.63%
**EfficientNet-B0**	**94.91%**	**95.55%**	**94.87%**	**94.90%**

*Note:* ↑ indicates that higher values represent better model performance.

## Data Availability

The experiment code and implementation files used in this study are available in the project GitHub repository: https://github.com/jamil226/turkish_food_analysis (accessed on 2 July 2026). The food image dataset used in this study is not available publicly due to privacy and ownership restrictions. However, we can provide the dataset via formal request to the authors made through an institutional email address.

## Abbreviations

The following abbreviations are used in this manuscript:

AIDCARE	AI-assisted Diabetes Care
AI	Artificial Intelligence
CNN	Convolutional Neural Network
CV	Computer Vision
DL	Deep Learning
ICR	Insulin-to-Carbohydrate Ratio
ISF	Insulin Sensitivity Factor
MAE	Mean Absolute Error
MLLM	Multimodal Large Language Model
MoH	Ministry of Health
RMSE	Root Mean Squared Error
